# Development and validation of a hybrid simulator for ultrasound-guided laparoscopic common bile duct exploration

**DOI:** 10.1007/s00464-023-10168-w

**Published:** 2023-06-16

**Authors:** Marine Y. Shao, Mohamed Aburrous, David Huson, Carinna Parraman, Jon Y. Hardeberg, James Clark

**Affiliations:** 1 Centre for Print Research, University of the West of England, Coldharbour Lane, Bristol, BS16 1QY United Kingdom; 2 Cornwall’s Centre for Healthcare Research and Innovation, Royal Cornwall Hospitals NHS Trust, Truro, TR1 3LJ United Kingdom; 3 Norwegian University of Science and Technology, N-2802 Gjøvik, Norway

**Keywords:** Surgical simulation, Common bile duct exploration, Laparoscopy, Validation

## Abstract

**Background:**

Ultrasound-guided laparoscopic common bile duct exploration (LCBDE) is the surgical management of choledocholithiasis. The procedure presents significant benefits to patients but still fails to be generalised because of the complex set of skills it requires. A simulator for ultrasound-guided LCBDE would allow trainee surgeons as well as experienced surgeons who perform this surgery seldomly to practice and gain confidence.

**Methods:**

This article presents the development and validation of an easily reproducible hybrid simulator for ultrasound-guided LCBDE which integrates real and virtual components of the task. We first developed a physical model made of silicone. The fabrication technique is replicable and allows quick and easy production of multiple models. We then applied virtual components onto the model to create training for laparoscopic ultrasound examination. Combined with a commercially available lap-trainer and surgical equipment, the model can be used for training the fundamental steps of the surgery through the trans-cystic or trans-choledochal approaches. The simulator was evaluated through face, content, and construct validation.

**Results:**

Two novices, eight middle grades, and three experts were recruited to test the simulator. The results of the face validation showed that the surgeons found the model realistic visually and felt realistic when performing the different steps of the surgery. The content validation indicated the usefulness of having a training system to practice the choledochotomy, the choledochoscopy and stone retrieval, and the suturing. The construct validation highlighted the ability of the simulator to differentiate between surgeons with various levels of expertise.

**Conclusions:**

The hybrid simulator presented is a low-cost yet realistic model which allows the surgeons to practice the technical skills required for trans-cystic and trans-choledochal ultrasound-guided LCBDE.

Gallstones are a widespread medical problem; 15% of the population in the USA and 5.9% to 21.9% of the population in Europe are affected by gallstones at one point in their adult life [1]. Symptomatic gallstones account for a significant number of the emergency admissions to hospital [2], with many presenting either acutely in pain or jaundiced. Although the endoscopic option is widely employed to extract the bile duct stones prior to any surgical procedure to remove the gallbladder, i.e. Endoscopic Retrograde Cholangiopancreatography (ERCP) followed by laparoscopic Cholecystectomy, there is a growing trend towards simply extracting the gallstones from the bile duct at the same time as surgery through a Laparoscopic Cholecystectomy and Common bile duct exploration (LCBDE) [3].

The keyhole, or laparoscopic, approach has significant benefits to the patients in terms of length of stay in hospital, blood loss, wound pain, and return to normal activities [4–6]. It is also recognised that to bring patients back for a second procedure as an outpatient, or for patients to remain as inpatients waiting for both procedures to be completed, is of far less preference than a single procedure with what can be an overnight stay or even as a daycase. However, the technique is laparoscopically challenging and requires multiple steps.

Surgical simulators are proven to improve a surgeon’s performance through operative rehearsal and practice [7]. This particular operation requires multiple skill sets to be undertaken in a series of well-executed steps including intra-operative ultrasound imaging of the bile duct as well as stitching using keyhole instruments [2]. This study describes the creation and subsequent validation of a reproducible hybrid surgical simulator for ultrasound-guided LCBDE.

This paper first presents the proposed materials and methods used to develop a hybrid simulator for LCBDE and evaluate it. The following sections provide a description of the results of the evaluation and the discussion.

## Materials and methods

The development of the hybrid simulator included multiple aspects. The first of these was the development of organ moulds using the Computer-Aided Design (CAD) software Rhino 7 (Robert McNeel & Associates, Seattle, USA). These moulds were 3D printed and used to pour silicone to obtain organ replicas. The silicone organs were assembled inside a commercially available lap-trainer to create a physical model.

The second aspect was the development of an augmented reality-based ultrasound training system. This training system was based on the display of virtual components onto the silicone model. These virtual components were ultrasound images that had been pre-recorded on a gel-based model. The choice of displaying ultrasound images through marker tracking instead of direct scanning of the silicone model was made for two reasons: (1) the silicone did not have the same acoustic properties as the soft tissues and for this reason it was not possible to directly record the silicone model; (2) using pre-recorded ultrasound images allowed training for laparoscopic ultrasound without having access to a laparoscopic ultrasound probe.

### Development of a physical simulator

An organ library was purchased online (Plasticboy Pictures CC, Cape Town, South Africa). This library included STL files of the soft tissues which were used as a basis to create the moulds of the soft tissues. These moulds were designed on Rhino using a Boolean difference between a rectangular block and the organ 3D model.

The moulds designed on Rhino were 3D printed. The printing technologies selected are Fused Deposition Modelling (FDM) and Stereolithography (SLA) because of the cost of the printers and of the cost of the printing materials [8]. In this project, we used the following 3D printers: the SLA printer Form 3 + (Formlabs Ohio Inc., Millbury, USA), and the FDM printers LulzBot TAZ Workhorse (LulzBot®, Fargo, USA) and Ender-5 S1 3D Printer (Creality, Shenzhen, China).

**Fig. 1:**
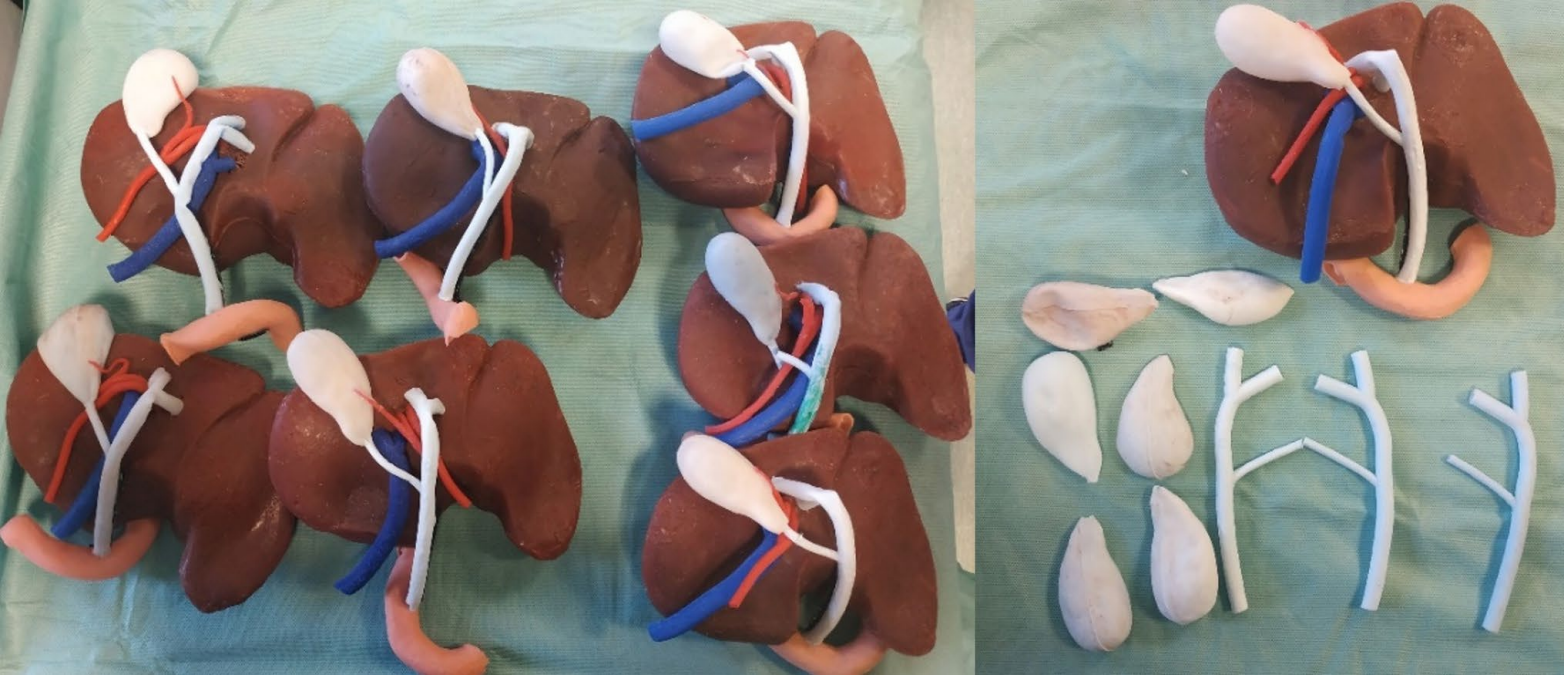
Development of multiple identical models for the training (left); Creation of replacement parts (right)

The benefits of using 3D-printed moulds are that the method is replicable and allows the creation of multiple models with the same anatomy, as shown in Fig. 1. This method is also convenient to create replacement parts for the synthetic soft tissues that get damaged during the simulation practice.

Silicone from Smooth-On (Smooth-On, Macungie, USA) was poured into the moulds to generate synthetic soft tissues. The liquid silicone was mixed with the pigments Silc Pig (Smooth-On, Macungie, USA) in order to recreate the appearance of the soft tissues.

The type of silicone used to mimic each soft tissue depended on its tactile properties and on how it feels during cuts and sutures. Table 1 summarises which material is used for the fabrication of the synthetic soft tissues.

**Table 1:** Materials used to mimic the soft tissues in the simulation

Soft tissue	Material	Price of the material (for 910 grammes)	Price for one part
Duodenum	Ecoflex 0030	£39.64	£2.2
Fat	Ecoflex gel	£39.05	£58
Skin	Ecoflex 0030 and stretchy fabric	£39.64	£24
Muscle	Ecoflex 0030 and cotton fibres	£39.64	£30
Gallbladder	DragonSkin	£43.48	£1
Bile duct	DragonSkin 75%-part A	£43.48	£1
Vein	DragonSkin 75%-part B	£43.48	£1
Artery	DragonSkin 75%-part B	£43.48	£0.5
Liver capsule	DragonSkin	£43.48	£1
Liver	Ecoflex gel 75%-part B	£39.05	£52

### Assembly of replica organs inside the lap-trainer

**Fig. 2:**
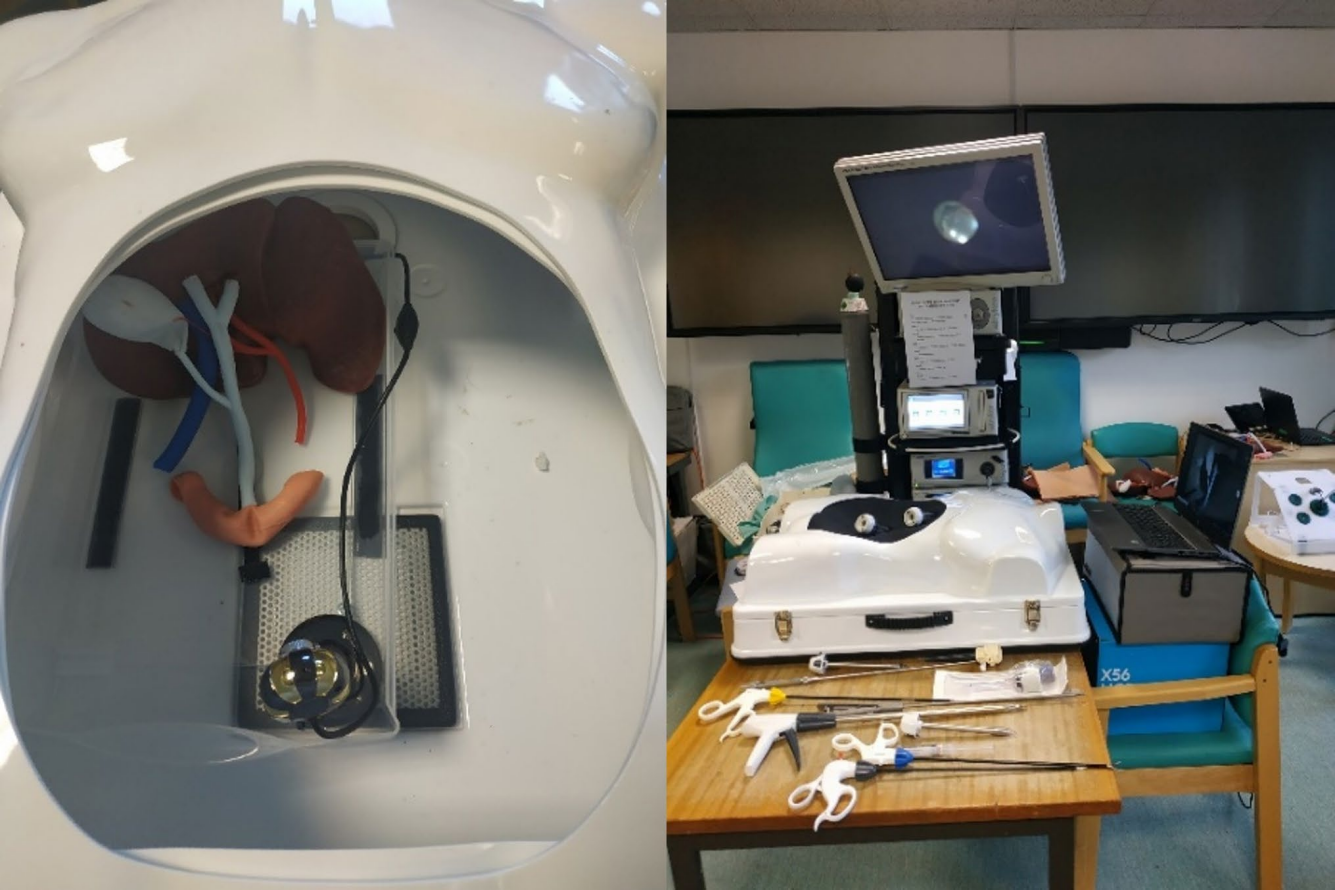
Setup of the synthetic soft tissues into the lap-trainer

The soft tissue replicas were sewn together using elastic yarn or glued with silicone glue (Smooth-On, Macungie, USA). The model was placed inside a commercially available box trainer (Erler-Zimmer, Lauf, Germany) and fastened using Velcro tapes. The soft cover was replaced with the simulated abdominal wall for the port insertion step then placed back on the lap-trainer for the remaining steps. A USB camera (Laparoscopic Training HD Camera, Gerati Healthcare Ltd, Sialkot, Pakistan) was placed inside the box trainer to record the scene using the Camera application (Microsoft Windows, Redmond, USA). The surgeons were also provided with the following laparoscopic instruments: Maryland, graspers, clip applier, choledochotomy knife, scissors, choledochoscope, Dormia basket, needle holders, and sutures. The setup is shown in Fig. 2.

### Augmented reality component

**Fig. 3:**
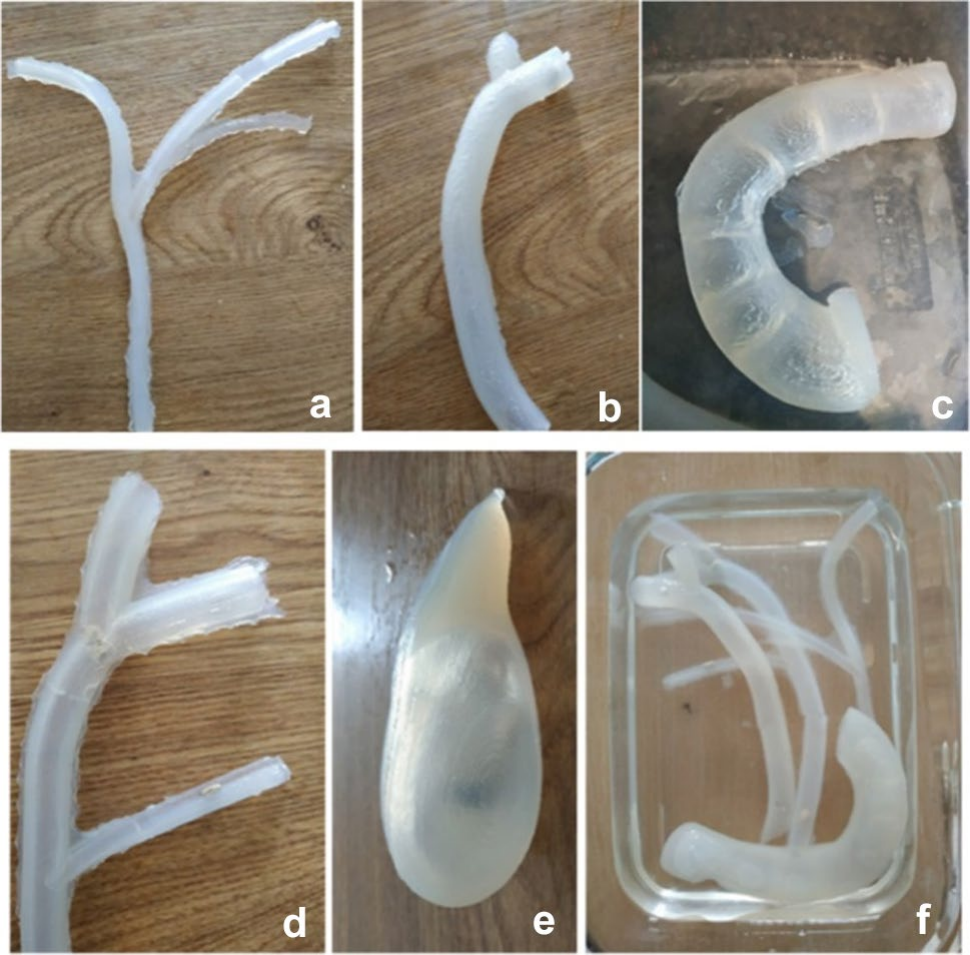
Fabrication of synthetic soft tissues made of agar gel

**Fig. 4:**
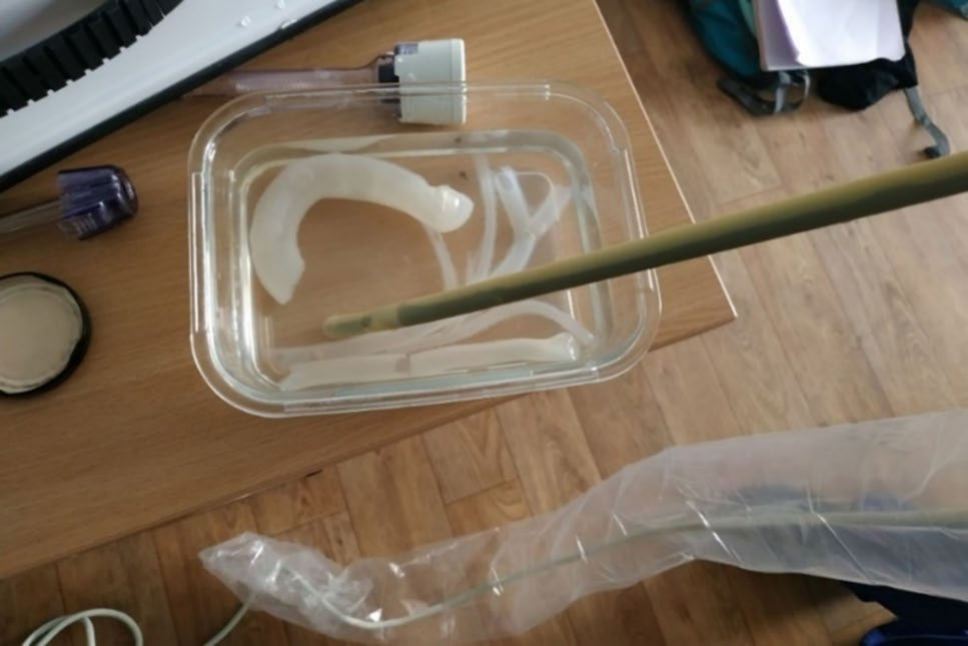
Recording of the ultrasound images on the agar model using a laparoscopic ultrasound probe

To create an augmented reality-based ultrasound examination, ultrasound images were pre-recorded using a laparoscopic ultrasound probe. The images were recorded on a model made of agar gel, as shown in Figs. 3 and 4. The model was cast using the same moulds as the silicone model in order to get the same anatomy. Simulated stones were included in the agar model. Different surgical scenarios were implemented by putting the simulated gallstones at different positions within the model. The positions of the stones were (1) next to the ampulla of Vater, (2) in the proximal bile duct, (3) in the hepatic duct, (4) in the cystic duct, and (5) in the gallbladder. The first three scenarios are indications for LCBDE.

The images were recorded from the end of the bile duct to the beginning of the hepatic duct by capturing short videos while moving the probe regularly. The ultrasound dataset is completed with videos of the cystic duct and of the gallbladder. The videos are recorded in B-mode.

**Fig. 5:**
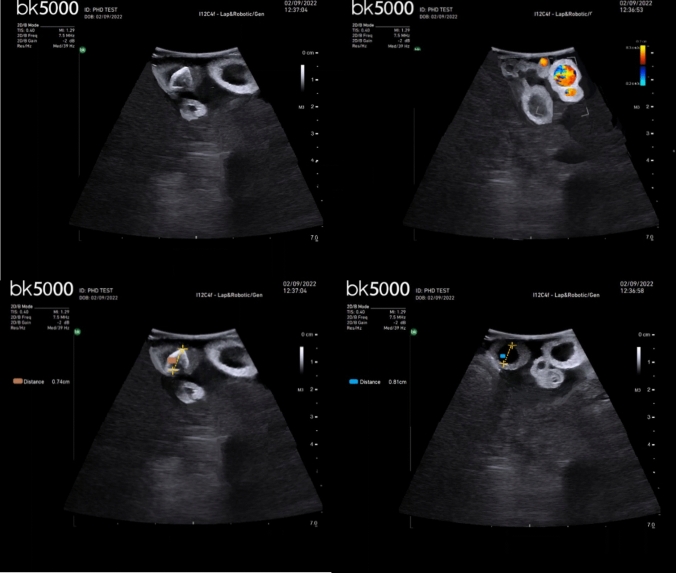
Creation of a dataset of ultrasound images in B-mode (upper left), Doppler mode (upper right), and with measures of the anatomical features (lower left and right)

The ultrasound images dataset was extracted from the videos and post-processed with the software Paint software (Microsoft Windows, Redmond, USA) to remove the echo recorded on the original images. Because the videos were only recorded in B-mode, the images were also post-processed to generate simulated Doppler images, as well as to get images with measures of the diameters of the bile duct and of the stones, this is shown in Fig. 5.

**Fig. 6:**
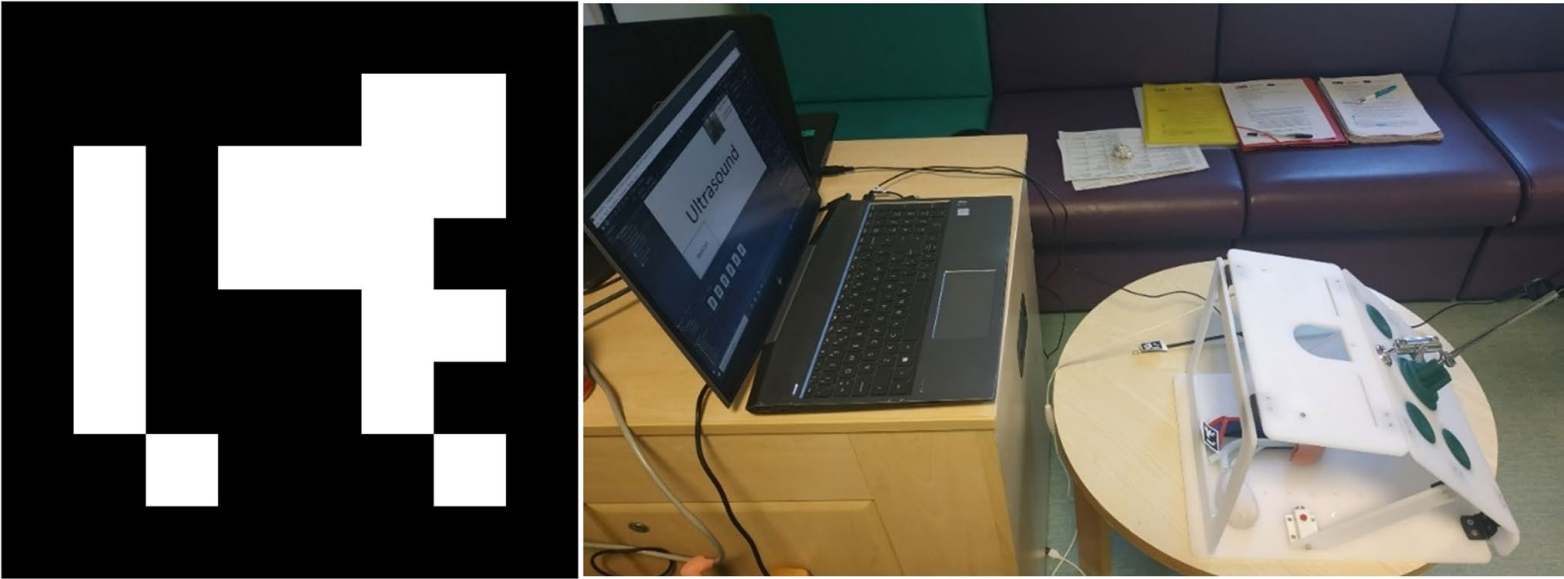
ArUco marker (left); setup for the augmented reality ultrasounds training (right)

The tracking of the marker was performed with Unity 2020 LTS, Visual Studio, Visual Studio Code, and Python 3.9. The setup of the system is shown in Fig. 6. With this setup, the endoscopic camera was connected to the computer and to the AR software. The ArUco markers were placed, respectively, on the simulated tissues and on the laparoscopic tool. The endoscopic camera recorded the live scene and detected the ArUco markers. ArUco markers are a type of fiducial markers used in context-aware augmented reality applications for the tracking of elements in the reality, as explained in [9]. Once the markers were detected, then the code could update the positions of the probe and the synthetic soft tissues in the simulated space [10] and the user could start the ultrasound training.

When the ultrasound images were displayed on the screen, the choice of the ultrasound image to display depended on the distance between the two markers. If $L$ is the distance between the liver and the duodenum in the simulated place, $n$ is the number of ultrasound images [*i*_*1*_, *i*_*2*_, …, *i*_*n*_] and $l$ the distance between the two markers, then the number of the images being displayed $i$ is described as

$$i=\frac{l }{L}\times n$$

### Evaluation

Surgeons were recruited under a University of the West of England Ethics Committee-approved protocol to perform simulated procedures on the model. The evaluation included face, content, and construct validation of the developed simulator.

During the training sessions, the surgeons were asked to perform the steps described in Table 2.

**Table 2:** Steps performed on the simulator

Step number	Description of the step
1	Port insertion
2	Clipping and division of the cystic artery
3	Milking, clipping, and partial transverse incision of the cystic duct
4	Ultrasound evaluation using the AR system; during this step, the surgeon has to assess the number of stones and locate them
5	From the ultrasound scan, the surgeon then decides the approach to common bile duct exploration: trans-cystic or trans-choledochal
6	Choledochotomy
7	Choledochoscopy and stone extraction
8	Suturing of the common bile duct

During the AR ultrasound training task, the surgeon could select from training modes 1 to 4. Each training mode representing one of the surgical scenarios. However, the physical model was made with a stone located at the bottom of the bile duct, which is scenario 1. For this reason, during the choledochoscopy and the stone retrieval the surgeon could only practice for this scenario.

The surgeons were asked to complete a questionnaire. This questionnaire first focussed on the background of the surgeons to assess their experience in performing LCBDE. The aim was to divide the participants into groups according to their level of expertise. The different levels of expertise were defined as follows:

- Novices: participants who had performed fewer than 10 laparoscopic surgeries as primary surgeons in the past and had no experience with LCBDE (participated in the surgery less than 10 times)
- Middle grades: participants with experience with laparoscopic surgery as primary surgeon (more than 40), but limited experience with LCBDE (less than 10 times as primary surgeons)
- Experts: participants with extensive experience with laparoscopic surgery and LCBDE (more than 40 times as primary surgeon).

The simulator was then assessed through face, content, and construct simulation. The face validation evaluation consisted of the assessment of the realism of the simulator. Content validity aimed to evaluate the usefulness of the training tool. For face and content evaluation, the surgeons were asked to use the Likert scale from 1 (poor performance), 3 (neutral), to 5 (excellent performance) to evaluate the model [11].

The construct validity aimed to demonstrate that the training system can differentiate between surgeons with different levels of expertise. For this study, it was implemented by comparing the completion time of the procedure, the assistance level (AL) in the completion of the tasks, the success in completing the tasks included in the simulator, and the number of instrument exchanges (IE). The evaluation also includes a total score (S) calculated as

$$S = \frac{{Time}}{{min\left( {max\left( {Time} \right),\,time\,limit} \right)}} + \frac{{AL}}{{max\left( {AL} \right)}} + \frac{{Number\,of\,failed\,tasks}}{{Number\,of\,tasks}} + \frac{{IE}}{{max\left( {IE} \right)}}$$

where Max score indicated the maximum score recorded among all participants. There was a time limit set at 10 min per task. If a user took more than 10 min, they were asked to stop the task; then the time taken was considered to be 10 min. For the tasks where every participant managed the task under the time limit, then the Max time is the time taken by the slowest participant, otherwise, the Max time was the time limit of 10 min.

The assistance level for the completion of the training tasks was evaluated using the Likert scale. The evaluation was carried out by a surgeon. The different assistance levels were the following:

1. No assistance (completed the task on their own with no indication),
2. An assistant surgeon held a grasper to maintain the soft tissues in place while the participant completed the task,
3. The participant received concise indications on how to perform the task combined with a high level of assistance given (an assistant surgeon handled some of the instruments in the task),
4. The participant received extensive explanations of the task, of how to perform it, and of how to use the tools, then a high level of assistance given (an assistant surgeon handled some of the instruments in the task), and
5. The assistant surgeon completed the task for the participant.

The difference in scores between the experts, middle grades, and the novices was statistically evaluated using the Kruskal–Wallis test in SPSS software.

## Results

**Fig. 7:**
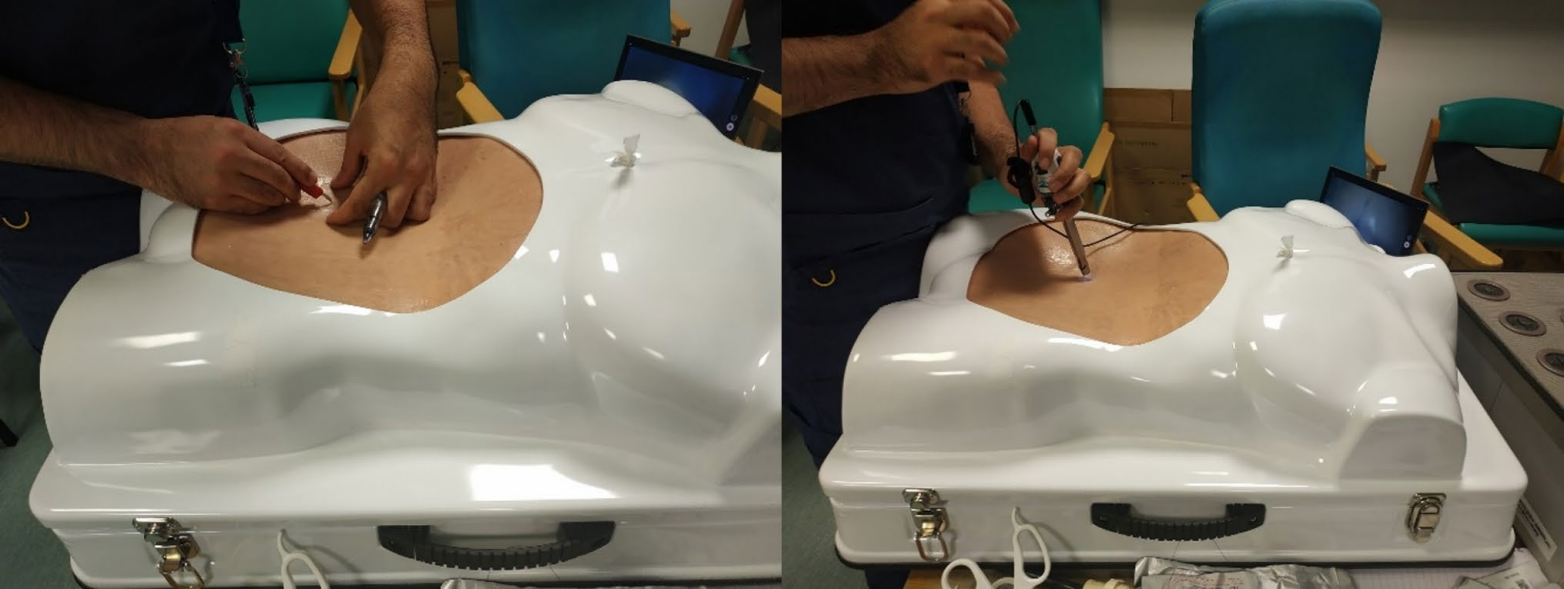
Training for port insertion

**Fig. 8:**
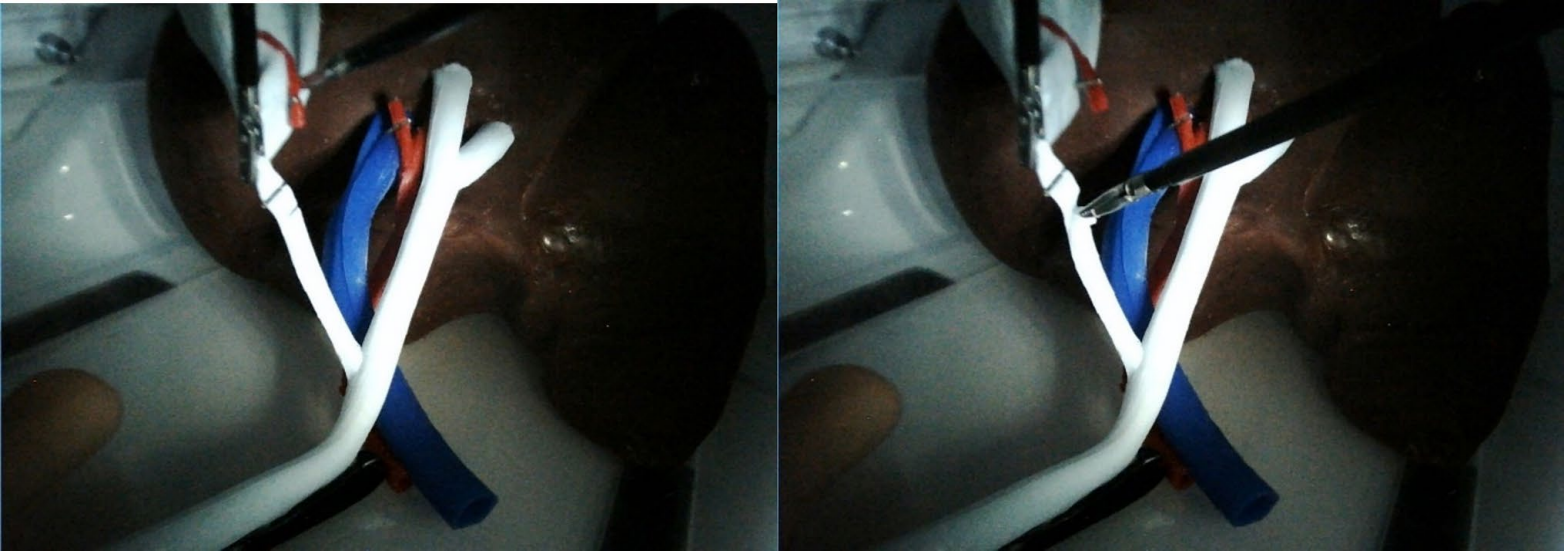
Clipping and dividing the cystic artery (left); milking, clipping, and partial division of the cystic duct (right)

**Fig. 9:**
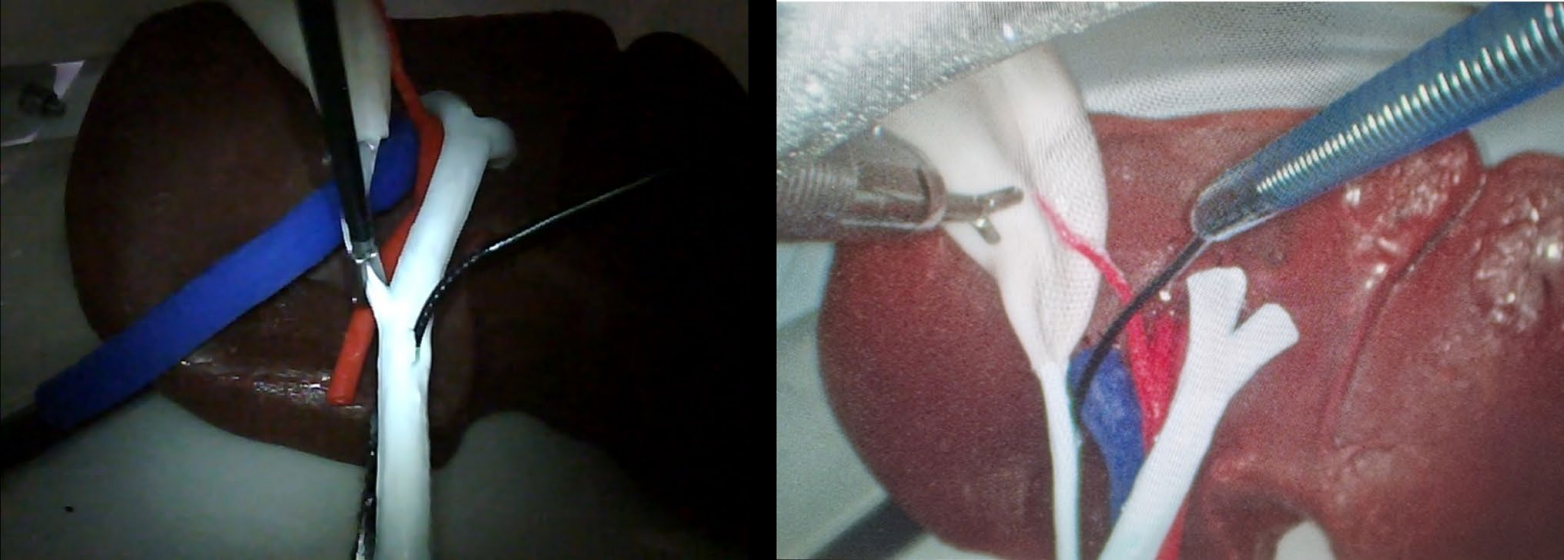
Insertion of the choledochoscope with a trans-choledochal approach (left) or a trans-cystic approach (right)

**Fig. 10:**
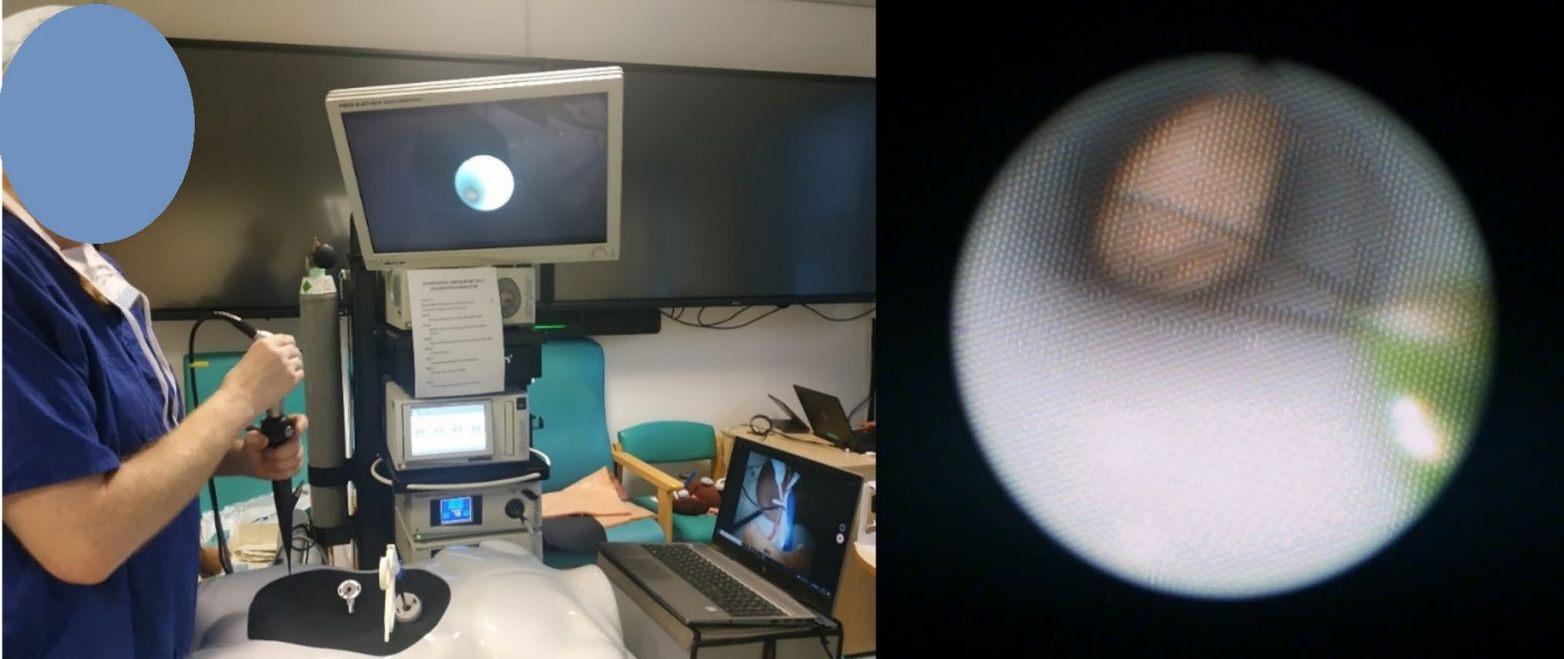
Choledochoscopy and capture of the stone

**Fig. 11:**
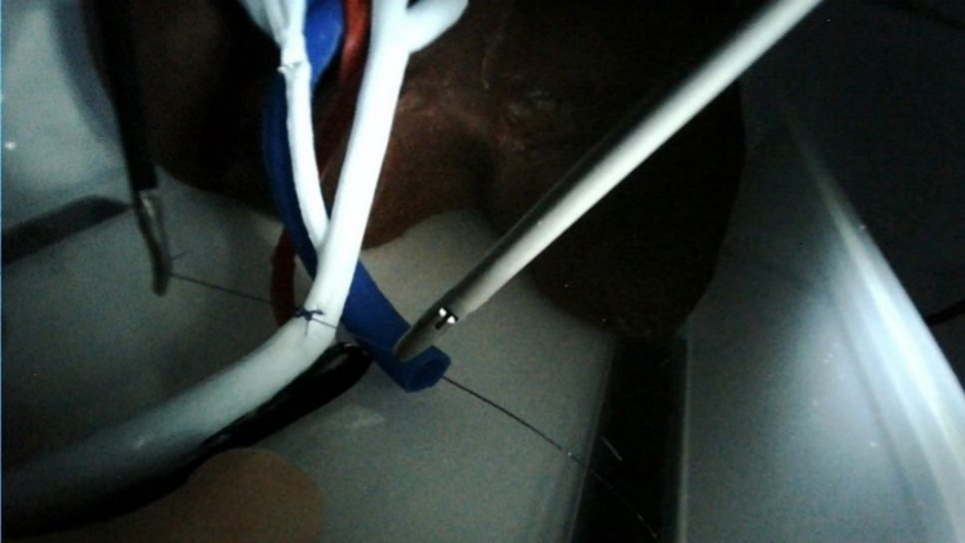
Suturing of the bile duct

Two novices, eight middle grades, and three experts were recruited to test the simulator. All participants were introduced to the model and to the aim of the simulator before starting a simulation practice. The steps of the simulated procedure were detailed before starting and during the course of the simulation if needed. The steps of the simulation are shown in Figs. 7, 8, 9, 10 and 11. The participants could all decide whether to use the trans-cystic or trans-choledochal approach.

A surgeon served as an assistant during the simulated procedure to help the participant. The surgeon could also give indications or explanations of the steps when required. This surgeon also evaluated the level of assistance required by each participant for the construct validation.

Table 3 details the results of the face validation of the simulator.

**Table 3:** Results of the face validation for the thirteen participants (mean score and standard deviation)

Visual realism	
Abdominal wall	4.3 ± 0.5
Liver	4.4 ± 0.5
Artery	3.4 ± 0.7
Vein	3.8 ± 0.8
Bile duct	3.4 ± 0.5
Gallbladder	4.1 ± 0.7
Duodenum	4.1 ± 0.7
Tactile realism	
Abdominal wall	4.1 ± 0.7
Liver	4.1 ± 0.7
Artery	3.4 ± 1.1
Vein	3.2 ± 1.0
Bile duct	3.6 ± 0.7
Gallbladder	4.0 ± 1.1
Duodenum	4.0 ± 0.7
Realism of a task	
Port insertion	3.4 ± 0.7
Ultrasound images (appearance)	3.6 ± 0.9
Choledochotomy	3.6 ± 0.8
Choledochoscopy	4.4 ± 0.8
Stone retrieval	4.5 ± 0.9
Suturing of the bile duct	4.1 ± 1.0
Perceived utility of the task	
Port insertion	4.0 ± 0.6
Ultrasound scan	4.0 ± 1.3
Choledochotomy	4.4 ± 0.8
Choledochoscopy	4.7 ± 0.5
Suturing of the bile duct	4.4 ± 0.9
General questions	
Usefulness of having multiple scenarios	3.9 ± 1.1
Would they include the simulator in their training?	4.5 ± 0.5
Challenge of the simulation	4.5 ± 0.8

Most of the surgeons considered that the model included all the relevant soft tissues. However, one surgeon mentioned that it would have been useful to include the omentum. This surgeon was an expert with more experience and who would benefit from a more complex training.

The surgeons evaluated the realism of the port insertion with 3.4 ± 0.7 (between neutral and good) as they said that it was not tough enough to be very realistic; especially, the muscle layer was not tough enough. Two surgeons said that it would be useful to also have a fascia layer. The choledochotomy and the suturing received lower scores, 3.6 ± 0.8 and 4.1 ± 1.0, respectively; but this might be due to poor lighting and visualisation. In real surgery, the camera has a better resolution and is adjusted in real time by an assistant. The stone retrieval received the best score of 4.5 ± 0.9 for the realism of the task.

Table 4 details the results of the content validation of the simulator.

**Table 4:** Results of the content validation (mean score and standard deviation)

Confidence level		
Port insertion	Before	4.8 ± 0.4
	After	4.8 ± 0.5
Ultrasound scan	Before	3.3 ± 1.5
	After	3.5 ± 1.0
Decision of the approach	Before	3.0 ± 1.7
	After	3.8 ± 1.0
Choledochotomy	Before	3.5 ± 1.5
	After	3.6 ± 1.2
Choledochoscopy	Before	3.2 ± 1.6
	After	3.7 ± 1.3
Suturing of the bile duct	Before	3.2 ± 1.5
	After	2.5 ± 1.3
Usefulness of being trained for the task		
Port insertion		4 ± 0.7
Ultrasound training		3.9 ± 0.8
Decision of the approach		4.2 ± 0.8
Choledochotomy		4.5 ± 0.7
Choledochoscopy and stone retrieval		4.5 ± 0.7
Suturing		4.4 ± 0.7

The confidence level for the insertion of the port remained at 4.8 (mean score), which indicated that this was a basic task and that most surgeons did not need to practice it. The mean confidence level decreased for the suturing, but this could be due to the visualisation issue.

Four of the surgeons were not confident in knowing the steps of the surgery; however, they said that the simulator could teach the steps of the surgery with a score of 4.3 ± 0.7, which shows the ability of the simulation to teach the steps of the surgery to less confident surgeons.

The results on the usefulness of being trained for each task show that the surgeons mostly needed training for the choledochotomy, the choledochoscopy and stone retrieval, and the suturing.

For the construct validation, participant 3 was withdrawn from the results as they had tested the simulator before participating in the study. The analysis therefore included three experts, seven middle grades, and two novices.

In this analysis, the scores were the completion time of the procedure in seconds, the assistance level evaluated using the Likert scale from 1 (no assistance) to 5 (maximum assistance), the success in completing the tasks included in the simulator (succeed or fail), the number of instrument exchanges (IE), and the total score (S). A low score indicated a better performance.

The construct evaluation showed that the three experts succeeded in completing all the tasks they were assigned. The middle grades usually succeeded in completing most tasks, except the suturing or the stone retrieval for five of the participants. The two novices also succeeded in most tasks, but both failed the suturing task; they also required more assistance than the middle grades and experts. Table 5 details the results of the construct validation of the simulator.

**Table 5:** Results of the construct validation (mean score and standard deviation)

Evaluation criteria	Level	Score	p-value (below 0.05 indicates statistical significance)
Assistance level to complete the procedure	Novice	17.5 ± 2.1	*P* = 0.068
	Middle grade	9.3 ± 2.0	
	Expert	8 ± 2	
Time to complete the procedure	Novice	1681 s ± 224 s	*P* = 0.018
	Middle grade	1193 s ± 236 s	
	Expert	642 s ± 91 s	
Instrument exchanges	Novice	26.5 ± 0.7	*P* = 0.041
	Middle grade	22.1 ± 3.8	
	Expert	17.7 ± 1.2	
Total score (S)	Novice	2.9 ± 0.3	*P* = 0.019
	Middle grade	2.0 ± 0.3	
	Expert	1.4 ± 0.2	

Evidently surgeons did not evaluate the utility of being trained for the different tasks similarly, and their evaluations were dependent on their experience of the procedure. Surgeons who perform the LCBDE infrequently found it more useful to be trained in the choledochotomy (mean 4.9 ± 0.4 instead of 4.0 ± 0.7) (*p* = 0.012), identifying the simulator as more effective in teaching this step (mean 4.9 ± 0.4 instead of 0.8 ± 0.8) (*p* = 0.01). Similarly, surgeons who perform LCBDE infrequently usually found it more useful to be trained in the laparoscopic ultrasound element (mean 4.3 ± 0.5 instead of 3 ± 0) (*p* = 0.017) and found the simulator more effective in teaching this step (mean 5 ± 0 instead of 2.7 ± 0.6) (*p* = 0.016).

## Discussion

This article presents the development of a hybrid simulator for ultrasound-guided LCBDE. The model could be used to simulate the trans-cystic or trans-choledochal approaches of LCBDE as well as laparoscopic ultrasound examination. It was made from relatively inexpensive materials (silicone). We have demonstrated the potential of this model through face, content, and construct validation.

The laparoscopic technique used to manage common bile duct stones has definite advantages to patients but has not been widely adopted by surgeons because of the challenging nature of the surgery. One of the challenges is the undertaking of a laparoscopic ultrasound examination of the bile duct with many opting for mobile fluoroscopy. This is very time limiting and as such may put many surgeons off from undertaking the procedure. Laparoscopic ultrasound as part of the procedure is key to acceptance but with no recorded available simulator able to teach this step of the procedure within the relevance of the task. With this lack of a training option, surgeons often move to use animal models to practice. This leads to a number of issues surrounding the price, access, and ethics. The model developed and described in this article could provide an alternative, sustainable, and reproducible training method.

The simulator presented can provide effective training at a relatively low price. While the initial model including all the soft tissues and the simulated abdominal wall is quite expensive to make (£60 for the synthetic internal soft tissues and £110 for the abdominal wall), replacement parts can be made quickly and at a very low cost. At each training session, only the bile duct and the artery received cuts and needed to be replaced. Thanks to the replicable fabrication process, the new parts can be moulded and replaced in a few hours. The price of each replacement part is £0.5 and £1 for the artery and bile duct, respectively.

The comments from the participants highlight that the model is very good and that the limitations faced during the evaluation were due to lighting and camera quality, which resulted in poor visualisation. Consequently, there were lower grades for some of the items, such as the suturing task, which was made more difficult than in real life.

Thanks to the type of fabrication technique used to develop the simulator, it would be possible to create more diverse anatomies by modifying the design of the organs and soft tissues on Rhino. This would offer more variety in the training and would increase the challenge of the simulation for more experienced surgeons.

## Conclusion

This article presented the development and validation of a unique, reproducible, low-cost hybrid simulator for trans-cystic and trans-choledochal ultrasound-guided LCBDE. The physical model is made of silicone using 3D-printed moulds which enables rapid replication of multiple models and the easy replacement of damaged parts after each training session. The validation highlighted the potential of such a model to improve the education of surgeons for this challenging procedure.

## References

[1] Winder JS, Pauli EM, Hazey J, Conwell D, Guy G (2016). Common bile duct stones: health care problem and incidence. Multidisciplinary management of common bile duct stones.

[2] Beckingham I, Macutkiewicz C, Toogood G, Maynard N (2015) Pathway for the Management of Acute Gallstone Diseases. Association of Upper Gastrointestinal Surgery. Accessed Dec. 15, 2022. [Online]. Available: https://www.augis.org/Portals/0/Guidelines/Acute-Gallstones-Pathway-Final-Sept-2015.pdf

[3] Al-Ardah MI (2023). Index admission vs elective laparoscopic common bile duct exploration: a district general hospital experience over 6 years. Langenbeck’s Arch Surg.

[4] Helmy MZ, Ahmed AE (2018). Safety and efficacy of laparoscopic versus open surgery in management of common bile duct stones: experience at the Sohag University Hospital, Egypt. Int Surg J.

[5] Redwan AA, Omar MA (2017). Common bile duct clearance of stones by open surgery, laparoscopic surgery, and endoscopic approaches (comparative study). Egypt J Surg.

[6] Vagholkar K, Nachane S, Vagholkar S (2021). Comparative study between laparoscopic and open cholecystectomy (Study of 50 cases). Int J Med Rev Case Rep.

[7] Bernier GV, Sanchez JE (2016). Surgical simulation: the value of individualization. Surg Endosc.

[8] Garcia J, Yang ZL, Mongrain R, Leask RL, Lachapelle K (2018). 3D printing materials and their use in medical education: a review of current technology and trends for the future. BMJ Simul Technol Enhanc Learn.

[9] Shao MY, Vagg T, Seibold M, Doughty M (2022). Towards a low-cost monitor-based augmented reality training platform for at-home ultrasound skill development. J. Imaging.

[10] Garrido-Jurado S, Muñoz-Salinas R, Madrid-Cuevas FJ, Marín-Jiménez MJ (2014). Automatic generation and detection of highly reliable fiducial markers under occlusion. Pattern Recognit.

[11] Joshi A, Kale S, Chandel S, Pal D (2015). Likert scale: explored and explained. Br J Appl Sci Technol.

